# Impact of apolipoprotein Ε4 (*APOE ε4*) on neuroimaging outcomes in cognitively healthy midlife adults: A systematic review

**DOI:** 10.1016/j.nicl.2026.104035

**Published:** 2026-07-15

**Authors:** Robert C. Davis, Louisa E. Wood, Lily Wilkes, Hannah L. Chandler, Emma L. Anderson, Lucy V. Hiscox

**Affiliations:** a Cardiff University Brain Research Imaging Centre (CUBRIC), School of Psychology, Cardiff University, Maindy Road, Cathays, Cardiff CF24 4HQ, UK; b Cardiff University Brain Research Imaging Centre (CUBRIC), School of Physics and Astronomy, Cardiff University, Maindy Road, Cathays, Cardiff CF24 4HQ, UK; c Division of Psychiatry, University College London, Tottenham Court Road, London W1T 7NF, UK

**Keywords:** Apolipoprotein *ε4*, Alzheimer's disease, Risk stratification, Neuroimaging, Biomarkers, Dementia

## Abstract

**Background:**

The apolipoprotein E *ε4* (*APOE ε4*) allele is the strongest genetic risk factor for Alzheimer's disease (AD); however, its neurobiological impact assessed by neuroimaging outcomes during midlife, before the onset of cognitive impairment, has not been summarised. This systematic review synthesises evidence on neuroimaging differences associated with *APOE ε4*-carrier status in cognitively healthy midlife adults and evaluates whether AD-related risk can be detected across imaging modalities.

**Methods:**

A literature search was conducted up until November 2025 to identify studies reporting neuroimaging outcomes in healthy adults aged 30–60 years with known APOE genotype. Eligible studies employed positron emission tomography (PET) and/or magnetic resonance imaging (MRI) and reported outcomes stratified by *APOE ε4-*carrier status. Risk of bias was assessed using the ROBINS-E tool, and a narrative synthesis was performed.

**Results:**

Searches yielded 7786 articles, and 46 studies met the inclusion criteria. Nine studies used PET, forty used MRI, and three of these studies employed both modalities. Substantial heterogeneity in methods and outcome reporting meant that a meta-analysis was not feasible. PET evidence consistently identified greater amyloid deposition and lower glucose metabolism in midlife *APOE* ε4-carriers compared with non-carriers, while MRI findings most reliably indicate higher cerebral blood flow. In contrast, other MRI-derived structural, microstructural, functional, and metabolic findings were mixed and largely inconclusive.

**Conclusion:**

Cognitively healthy *APOE ε4-*carriers demonstrate measurable neurobiological differences as early as midlife, consistent with a preclinical vulnerability associated with genetic risk for AD. These findings highlight the importance of characterising *APOE*-related mechanisms during midlife to inform early detection strategies and the development of preventative interventions for AD.

## Highlights

• Review synthesises neuroimaging evidence of APOE ε4 effects in healthy midlife adults.
• Overall, strong evidence that *APOE ε4*-carriers possess higher amyloid-β burden, lower glucose metabolism, and higher cerebral blood flow.
• Structural and diffusion MRI findings are mixed and inconclusive.
• Methodological heterogeneity in neuroimaging limits robust inference on *APOE ε4* effects.

## 1 Introduction

Alzheimer's disease (AD) is a progressive neurodegenerative disorder that typically manifests in later life, with most individuals developing symptoms after the age of 65 years. In the United Kingdom, a diagnosis of dementia is suspected with evidence of cognitive impairment, often accompanied by behavioural changes or difficulties performing activities of daily living (NHS, 2026). Diagnostic assessment is supported by biomarkers of AD pathology, classified within the A/T/N framework according to the presence of amyloid-β deposition (A), tau neurofibrillary tangle formation (T), and neurodegeneration (N) (Jack Jr et al., 2016). These biomarkers are measured using amyloid-β or tau positron emission tomography (PET), blood- or cerebrospinal fluid-based assays, or structural magnetic resonance imaging (MRI) to examine the extent of atrophy and neurodegeneration. Together these methods allow identification of AD-specific pathology, biomarker progression over time, and in some cases, differentiate AD from other types of dementia (Henneman et al., 2009). However, these overt symptoms reflect advanced macrostructural degeneration and are likely preceded by disease-specific neurobiological alterations occurring years or decades before diagnosis (Jia et al., 2024; Johnson et al., 2023).

Evidence from clinical trials suggests therapeutic interventions initiated during symptomatic stages of AD confer minimal cognitive or structural benefit (Aisen et al., 2022). This has led to increasing emphasis on identifying AD-related pathology at earlier, preclinical stages, where interventions may be more effective in delaying or preventing disease progression. Accordingly, some clinical trials target preclinical or Mild Cognitive impairment (MCI) populations, evaluating the efficacy of treatments such as lecanemab in reducing amyloid-β deposition over 18 months (Dyck et al., 2023) or in preventing the onset of tau pathology before the appearance of any measurable cognitive deficits (Rafii et al., 2023).

Apolipoprotein E (APOE) is the most well-established genetic risk factor for sporadic AD and exists in three common isoforms: ε2, ε3, and ε4. The least common *ε2* isoform is thought to be neuroprotective and has been associated with reduced AD-related histopathology (Kim et al., 2022)*,* while the *ε3* allele is the most prevalent and generally considered risk-neutral. Previous literature associated the *APOE ε4*-allele with increased amyloid-β deposition (Morris et al., 2010), potentially mediated through impaired clearance via low-density lipoprotein receptor–related protein 1 (Tachibana et al., 2019), increased microglial immunoreactivity surrounding amyloid plaques (Rodriguez et al., 2014), and elevated levels of phosphorylated tau (Shi et al., 2017). Consistent with these mechanistic findings, the *APOE ε4* allele confers the greatest known genetic risk for AD; *APOE ε4*-homozygotes have a ∼ 40% higher likelihood of developing AD by 85 years compared with APOE ε3/ε3 carriers (Genin et al., 2011). Together, these findings suggest that *APOE ε4* influences multiple pathological pathways central to AD pathogenesis.

Neuroimaging techniques play a central role in characterising AD-related brain changes across the disease continuum. PET imaging enables quantitative assessment of radioligand binding to insoluble protein targets, most commonly amyloid-β (isoforms Aβ_40_ and Aβ_42_) (Ruan and Sun, 2023) and phosphorylated tau (p-tau) (Cho et al., 2016), providing *in vivo* measures of AD-specific pathology. Other PET tracers such as [^18^F]-Fluorodeoxyglucose (FDG) offer insight into cerebral glucose metabolism, that is frequently altered in dementia and neurodegeneration (Mosconi and McHugh, 2011). MRI allows for both structural and functional assessment of brain health. Conventional T1- and T2-weighted MRI can quantify brain atrophy, volumetric differences, and white matter hyperintensities, while advanced techniques such as diffusion-weighted imaging and magnetic resonance spectroscopy enable the investigation of microstructural integrity and neurochemical composition, respectively.

Previous systematic reviews have evaluated neuroimaging differences between individuals with AD (Dang et al., 2023; Graff-Radford and Kantarci, 2013; Radmard et al., 2025) or MCI (Harrison et al., 2020; Liu et al., 2015) and cognitively healthy controls. Several have also assessed differences between older individuals carrying at least one *APOE* ε4 allele and *APOE* ε3 homozygotes. However, to date, no systematic review has comprehensively evaluated neuroimaging outcomes associated with *APOE ε4-*carrier status specifically in asymptomatic individuals across midlife; a critical period during which preclinical AD-related changes may first emerge.

The present systematic review seeks to comprehensively evaluate the influence of the *APOE ε4-*polymorphism on neuroimaging outcomes among cognitively unimpaired, asymptomatic individuals during early midlife/midlife. Additionally, we evaluate the methodological quality and risk of bias of the included studies to determine the strength and reliability of the existing evidence.

## 2 Methods

This systematic review was conducted in accordance with the Preferred Reporting Items for Systematic Reviews and Meta-Analyses (PRISMA) statement (Page et al., 2021) and prospectively registered with PROSPERO (CRD42024628228).

### 2.1 Search strategy

Using the OVID system, MEDLINE, Embase, PsycINFO, Web of Science, and SCOPUS databases were searched for studies in December 2025. Full search terms can be found in the supplementary material, but broadly relate to four search themes: *APOE*, Alzheimer's disease OR dementia biomarkers, preclinical OR middle-aged, neuroimaging modality.

### 2.2 Inclusion and exclusion criteria

Original contributions were included if they met the following criteria (PICOS). Population: (1) Healthy human participants aged between 30 and 60 years ±5 years standard deviation. Exposures: (2) Genotyped for the *APOE ε4* polymorphism. Comparisons: (3) Individuals without the *ε*4 allele as a control group. Outcomes: (4) Imaging findings from derived measures of brain structure (e.g., MRI or other modalities such as PET). Study design: Cross-sectional and cohort studies were included. Exclusion criteria were as follows: (1) reviews, editorials, and letters not containing original data; (2) case reports or case series   < 10 participants; (3) adults diagnosed with neurological disease or disorder; (4) studies in which the mean participant age was outside 30 to 60 years, or where one standard deviation around the mean extended below 25 years or above 65 years; (5) non-English language publications.

### 2.3 Data extraction and literature quality assessment

**Fig. 1:**
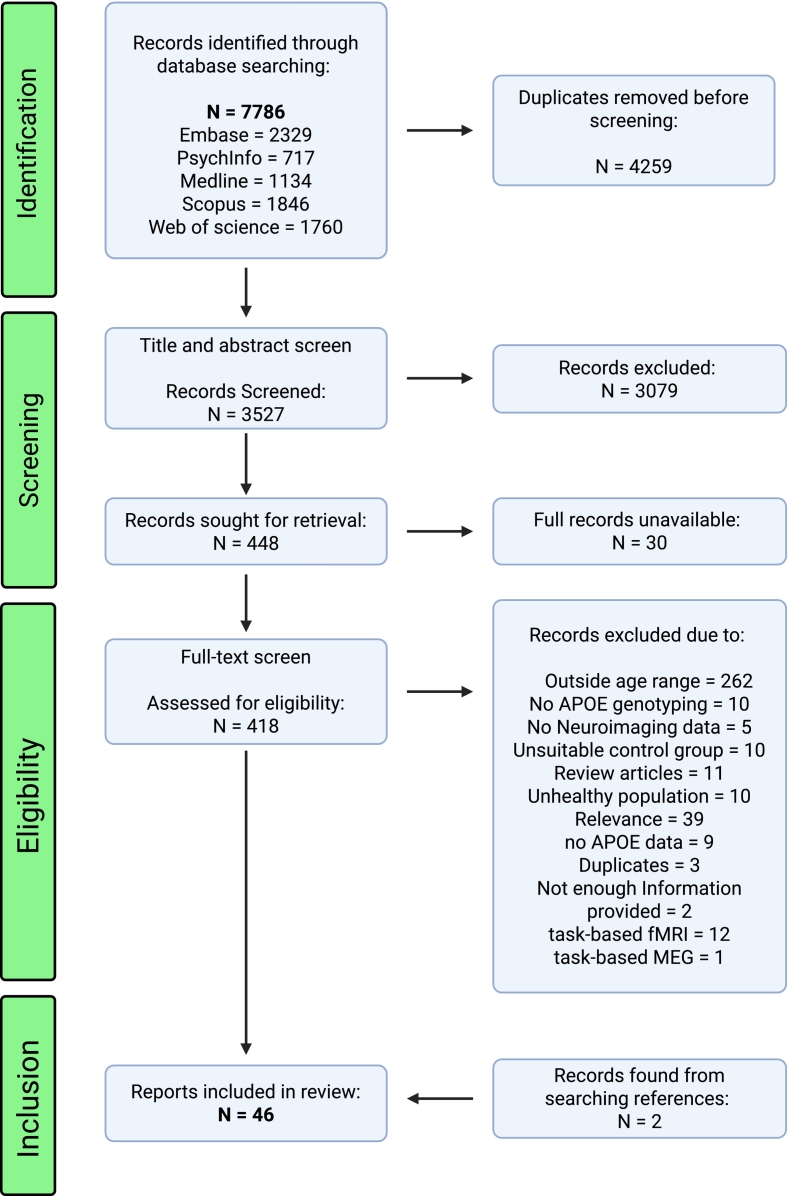
PRISMA flow diagram showing the identification, screening, eligibility assessment, and inclusion of studies in the systematic review.

All articles retrieved through the search were imported into Rayyan (Mourad Ouzzani et al., 2016). Duplicates were identified electronically, using inbuilt duplicate detection software in EndNote and later in Rayyan. Titles and abstracts were screened independently by three reviewers (RCD, LEW, LW), each blinded to the others' decisions. At this stage, 20% of records were additionally screened by a fourth reviewer (LH), yielding an inter-rater reliability of 80%. Any discrepancies following full-text screening were resolved through discussion. The study selection process is illustrated in Fig. 1.

Data extraction was conducted independently by all four reviewers using a standardised form. For 50% of all included papers, two reviewers extracted information and had discussions on the final material included. Extracted information included: First author, year of publication, institution, and country of the first author, sample size, mean age ± standard deviation, *APOE* genotype and how these groups were stratified (i.e. *APOE ε4-*carriers vs non-carriers or specific allele combinations), radiotracer for PET studies or field strength if MRI, and main findings. When available, *p*-values and effect sizes were also extracted.

When the initial search was formulated, the review aimed to synthesise all structural, diffusion, spectroscopic, functional, and vascular studies, while explicitly excluding task-based functional MRI. This was to reduce variability introduced by task performance and allow a clear focus on *APOE-*related effects on intrinsic brain structure and physiology.

A quality assessment was conducted with the ROBINS-E tool (Higgins et al., 2024). Each study underwent a methodological evaluation based on the six categories in the checklist, totalling twenty-four questions. Each question was scored as “Yes”, “No”, or “Unclear”. The primary exposure was *APOE ε4* allele status, aiming to compare *APOE ε4-*homozygotes, *ε3/ε4-*heterozygotes, and *ε3/ε3-*homozygotes. The main confounding factors assessed were cardiovascular risk factors (e.g. hypertension, diabetes, dyslipidaemia), and vascular conditions (e.g. history of stroke, atherosclerosis). We further examined whether cognitive health status was assessed to ensure participants were free of any signs of cognitive impairment. This systematic approach ensured that the methodological quality and risk of bias were consistently evaluated across all studies.

### 2.4 Strategy for data synthesis

A descriptive synthesis was undertaken to summarise and integrate the aggregate findings from the included studies, organised broadly by imaging modality (PET or MRI). The synthesis examined whether APOE genotype was associated with structural, physiological, and functional brain changes, alongside key study characteristics including the classification of *APOE ε4-*carrier status (e.g., homozygotes or heterozygotes), study design, sample size, sex distribution, imaging methods and reporting outcomes.

## 3 Results

The initial search identified 7786 articles, of which 4259 were removed as duplicates. During title and abstract screening, a further 3079 articles were excluded due to irrelevance to the study aims, leaving 448 for full-text review. Of these, 30 articles could not be retrieved, primarily being conference abstracts with no published full text or articles with inaccessible full text. The remaining 418 articles underwent eligibility assessment, resulting in the exclusion of 374 articles for reasons such as being outside the appropriate age range, lacking a suitable control group, or providing insufficient data. Ultimately, 46 articles met the eligibility criteria and were included in the final review (Fig. 1). Data extraction was performed as described in Methods and broadly categorised into PET and MRI studies.

### 3.1 Risk of Bias assessment

**Fig. 2:**
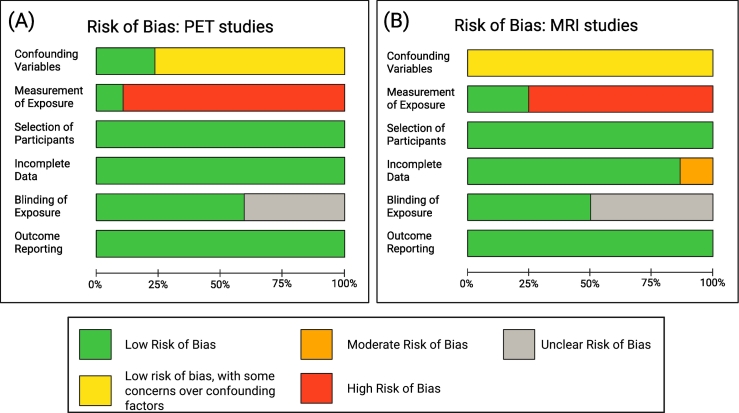
Risk of bias for (A) PET and (B) MRI studies.

The ROBINS-E tool for methodological quality assessment of epidemiological studies was used to quantify risk of bias across six domains: Confounding variables, measurement of exposure, selection of participants, incomplete data, blinding of exposure, and outcome reporting (Fig. 2). In the context of this review, ‘blinding of exposure’ refers to whether investigators involved in neuroimaging data acquisition and image processing were blinded to participants' *APOE* ε4-carrier status. This domain assesses the potential for knowledge of exposure status to influence the processing, assessment, interpretation, or analysis of neuroimaging outcomes. Studies were rated as having a low risk of bias when researchers were reported to be blinded to *APOE* ε4 status, a high risk of bias when investigators were reported to be aware of *APOE* ε4 status, and an unclear risk of bias when blinding procedures were not sufficiently described.

Studies aptly reported cohort demographic information and the criteria for inclusion/exclusion. The main confounding factors assessed were age, cardiovascular risk factors (e.g. hypertension, diabetes, dyslipidaemia), and vascular conditions (e.g. history of stroke, atherosclerosis). Cardiovascular risk factors or vascular conditions were described in 30 of the 46 studies (26 described cardiovascular risk factors, 21 described pre-existing vascular conditions). Cognitive health was assessed in 44 of the 46 studies using validated cognitive and memory assessments to reduce, though not eliminate, confounding from existing impairment. The measured exposure was the *APOE ε4-*allele, with most studies reporting reference single nucleotide polymorphisms (SNPs) and the methodology used to measure *APOE* status, primarily utilising TaqMan genotyping. The majority of studies (72%, *n* = 33) gave unclear information on how *APOE ε2* carriers were grouped or included *APOE ε2-*allele carriers in their analyses, but in insufficient numbers to perform a gene-dose analysis to improve biological interpretability. One study had incomplete data and excluded over a third of the cohort (553 individuals) due to “image corruption and/or postprocessing errors” (Dounavi et al., 2024).

Of the 46 studies identified, only one study was rated as having the lowest possible risk of bias according to the ROBINS-E tool (Mecca et al., 2018). This study provided appropriate covariate control with age- and sex-matched participants, restriction to specific APOE genotypes (*ε3/ε3, ε3/ε4, ε4/ε4*), comprehensive medical histories, multiple cognitive assessments confirming no memory impairment, blinding of experimenters to genetic status, and outcome reporting aligned with pre-specified hypotheses.

### 3.2 PET imaging

A total of 10 cross-sectional PET studies were identified. Six studies used [^18^F] fluorodeoxyglucose (FDG) PET to examine glucose metabolism (Reiman et al., 1996; Reiman et al., 2004; Reiman et al., 2005; Langbaum et al., 2010; Protas et al., 2013; Chen et al., 2012), two used [^11^C] Pittsburgh Compound B (C-PiB) to measure fibrillar amyloid burden (Mecca et al., 2018; Thirunavu et al., 2019), and two studies employed both radiotracers.

Most PET studies had limited sample sizes (from 27 to 250; see Table 1). The mean age across PET studies was 52 years; eight studies reported mean ages between 53 and 59 years, and two studies reported considerably lower means (31 and 42 years). *APOE ε4*-carrier status was defined inconsistently: Some included only *APOE ε4* heterozygotes, while others combined heterozygotes and homozygotes into an *ε4* carrier group. Control groups also differed, with a few studies restricting controls to *APOE ε3* homozygotes, whereas others did not report the allele composition of their non-carrier samples (Thirunavu et al., 2019).

**Table 1:** PET study characteristics.

Author	Country	Sample Size	Age (±SD)	Genotype	Radiotracer	Main Finding
Glucose Metabolism						
(Reiman et al., 1996)	USA	33	56.0 (4.5)	1,3	[^18^F] Fluorodeoxyglucose	ε4 homozygotes had lower glucose metabolism in posterior cingulate, parietal, temporal, and pre frontal cortex regions (*z =* 4.01–6.58)*.*
(Reiman et al., 2004)	USA	27	31.0 (5.1)	1,2	[^18^F] Fluorodeoxyglucose	ε4 heterozygotes had lower glucose metabolism in posterior cingulate, parietal, temporal, and pre frontal cortex (*p =* 0.008–0.049).
(Reiman et al., 2005)	USA	160	56.2 (4.7)	2,3,b	[^18^F] Fluorodeoxyglucose	ε4 gene dose effect showing lower glucose metabolism in posterior cingulate, precuneus, and left parietotemporal region after correcting for multiple comparisons. (*p <* 0.05).
(Langbaum et al., 2010)	USA	27	54.6 (6.2)	a,b	[^18^F] Fluorodeoxyglucose	Latino ε4 carriers had lower glucose metabolism than non-carriers in posterior cingulate, precuneus and parietal regions; also lower metabolism in middle and anterior cingulate cortex, hippocampus and thalamus (*p <* 0.05).
(Chen et al., 2012)	USA	130	56.2 (4.7)	2,3,b	[^18^F] Fluorodeoxyglucose	ε4 carriers had lower glucose metabolism in posterior cingulate, parietal, temporal, and pre frontal cortex (*p* < 0.05).
(Protas et al., 2013)	USA	149	56.2 (4.6)	1,2,b	[^18^F] Fluorodeoxyglucose	ε4 gene dose was correlated with lower glucose metabolism in the posterior cingulate cortex and hippocampus (*r* = 0.29*, p =* 0.0003).
(Bussy et al., 2019)	USA	162	57.1 (5.5)	2,b	[^18^F] Fluorodeoxyglucose	No main effect of APOE on glucose metabolism, or × age interaction.
(Stonnington et al., 2020)	USA	114	55.6 (5.2)	a,b	[^18^F] Fluorodeoxyglucose	Lower glucose metabolism in ε4 carriers compared with non-carriers in mainly parietal regions (*p* < 0.001).

Amyloid deposition						
(Mecca et al., 2018)	USA	45	59.1 (4.7)	1, 2, 3	[^11^C] Pittsburgh Compound B	ε4 homozygotes had higher cortical Aβ burden than ε3 homozygotes. Higher Aβ in frontal cortex, lateral temporal, lateral parietal, posterior cingulate, and precuneus (*p =* 0.003).
(Bussy et al., 2019)	USA	162	41.4 (11.4)	2,b	[^11^C] Pittsburgh Compound B	Significant interaction between age and APOE status on amyloid deposition in both precuneus and pooled cortical regions, however this effect could be driven by age (*p* < 0.05).
(Thirunavu et al., 2019)	USA	96	53.5 (45–60)	a,b	[^11^C] Pittsburgh Compound B	ε4 carriers had elevated cortical Aβ burden (Centiloid units) compared to non-carriers. ROI included prefrontal cortex, precuneus, gyrus rectus, and lateral temporal regions (β = 6.1, *p* = 0.0012).
(Stonnington et al., 2020)	USA	58	56.6 (4.3)	a,b	[^11^C] Pittsburgh Compound B	ε4 carriers showed higher amyloid deposition in mainly frontal regions (*p* < 0.001).

1 = ε3 homozygotes; 2 = ε4 heterozygotes; 3 = ε4 homozygotes; a = ε4 carriers; b = ε4 non-carriers.

#### 3.2.1 Glucose metabolism

**Fig. 3:**
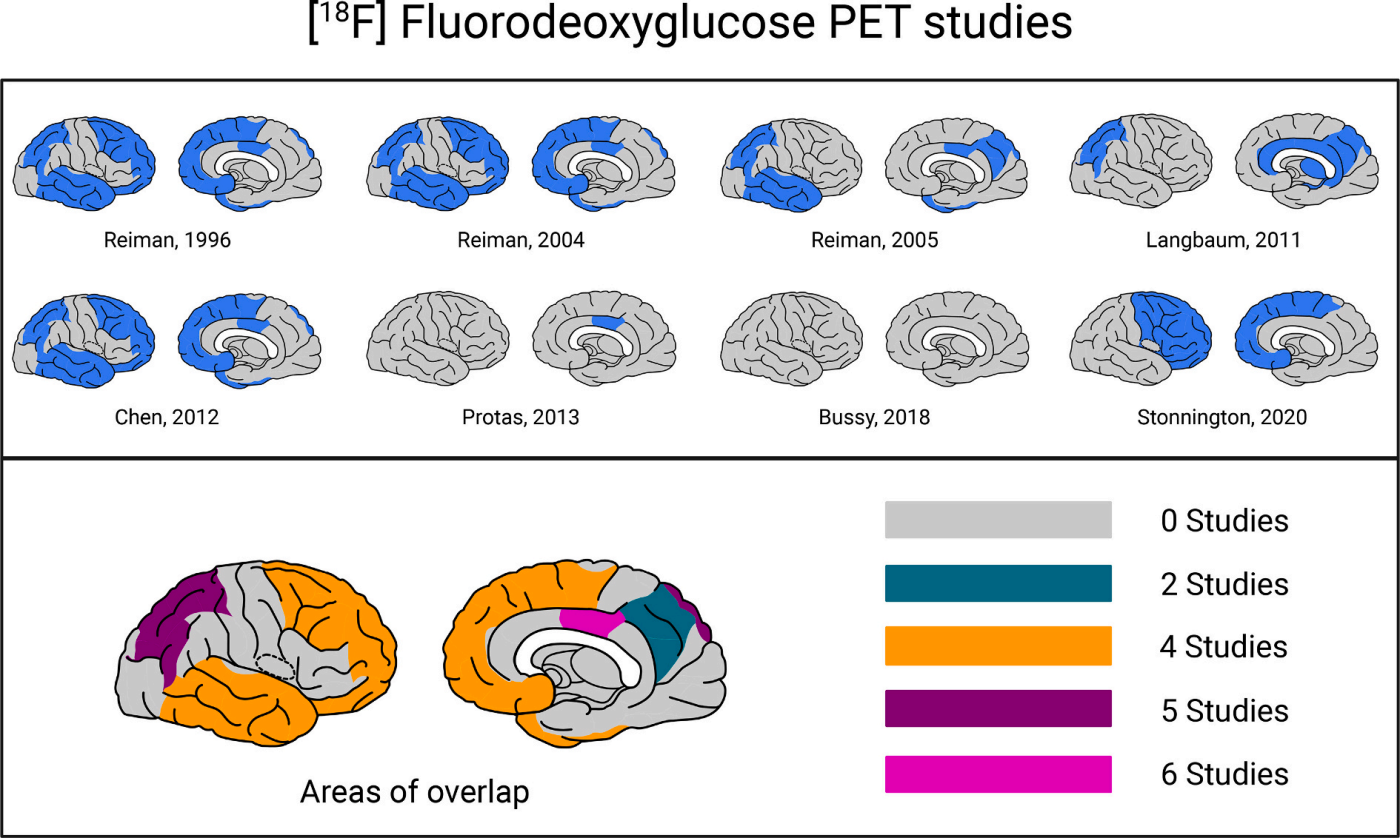
Fluorodeoxyglucose (FDG) uptake was lower (blue) in seven of the eight studies, implicating multiple brain regions affected by APOE ε4 carriership. Areas of overlap included the posterior cingulate cortex (6 studies; pink), parietal regions (5 studies; purple), frontal and temporal regions (both in 4 studies; orange), and the precuneus (2 studies; teal). Brain maps were created using Simple brain plot (Scholtens et al., 2021).

Eight studies examined differences in cerebral glucose metabolism measured using [18F]-fluorodeoxyglucose (FDG), between *APOE ε4-*carriers and non-carriers (Fig. 3). Six studies quantified FDG uptake as milligrams per minute per 100 g of tissue, while two reported standardised uptake value ratios (SUVR). Regions of interest included areas involved in memory processing and default mode network (DMN) function, such as the precuneus, hippocampus, posterior and anterior cingulate, parietal, temporal and prefrontal cortices. The majority of studies reported lower glucose metabolism in *APOE ε4*-carriers compared with non-carriers, most consistently affecting parietal and posterior cingulate regions. Although the direction of effect was consistent, the magnitude and regional specificity varied across studies, likely reflecting methodological variability.

Early evidence comparing *APOE ε4-*homozygotes with *APOE ε3-*controls reported reduced glucose metabolism in the posterior cingulate cortex (PCC), parietal, temporal, and prefrontal regions (*z-*score: 4.01–6.58) (Reiman et al., 1996). Subsequent evidence, from a younger, smaller cohort of *APOE ε4-*carriers (*n* = 27) similarly reported lower metabolic rates in the same regions (*p =* 0.008–0.049) *(*Reiman et al., *2004**)*. In two larger cohorts (*n* = 160 and *n* = 130) which compared homozygotes, heterozygotes, and non-carriers, hypometabolism was reported in the PCC (Reiman et al., 2005; Chen et al., 2012), left parietotemporal cortex (Reiman et al., 2005), precuneus (Reiman et al., 2005), prefrontal, temporal, and parietal cortices (*p* < 0.05) (Chen et al., 2012).

Further support for region-specific vulnerability was provided by a study examining cerebral metabolic rate of glucose (CMRgl) in the hippocampus and PCC, identifying a negative *APOE ε4* gene-dose effect. Specifically, each additional *APOE ε4* allele was associated with progressively reduced CMRgl in the PCC (*p =* 0.0003) (Protas et al., 2013), however, no differences were observed in the hippocampus. One small study in a Latino cohort reported reduced glucose metabolism across multiple regions, including the PCC, precuneus, parietal cortex, mid- and anterior cingulate, hippocampus, and thalamus, with effects persisting after partial-volume correction (*p <* 0.05) (Langbaum et al., 2010).

In contrast, one study reported no differences in glucose metabolism between *APOE ε4-*carriers and non-carriers (Bussy et al., 2019). These findings may reflect true null effects but may also be influenced by a different study design, including the relatively younger age of the population (mean age = 41.4 years) and the exclusive selection of the precuneus. The only longitudinal study, with a 7-year follow-up, observed lower baseline frontal region glucose metabolism in ε4-carriers (*p* < 0.001) but no *APOE*-specific longitudinal decline; instead, metabolic changes were influenced by brain-derived neurotropic factor (BDNF) genotype, and no main *APOE* effect could be determined (Stonnington et al., 2020).

Overall, glucose metabolism is consistently lower in *APOE-ε4* carriers than in non-carriers, with strong regional concordance in the PCC and parietal, temporal, and frontal cortices.

#### 3.2.2 Amyloid burden

**Fig. 4:**
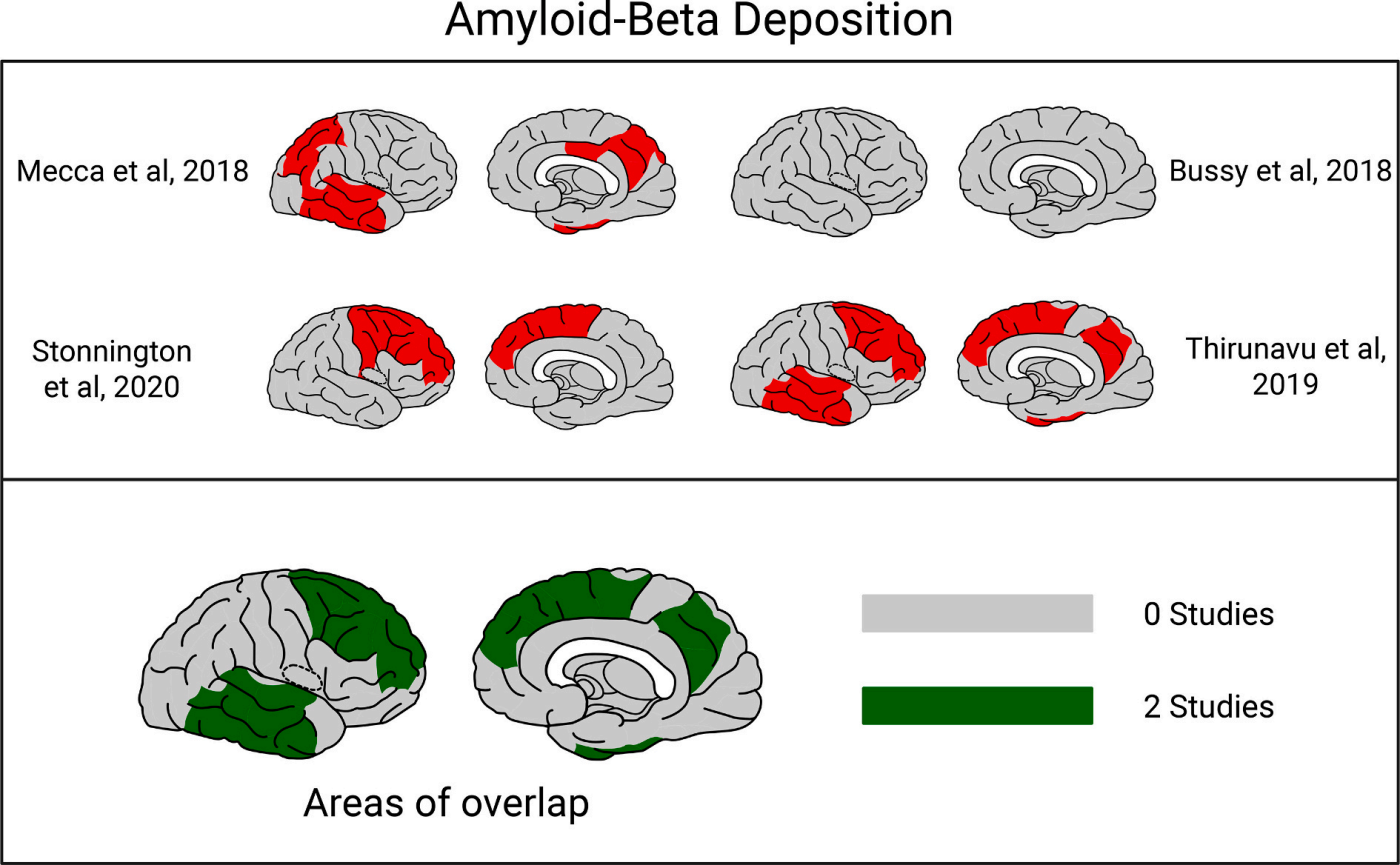
Three of four studies reported higher cortical amyloid-β deposition in APOE ε4-carriers compared with non-carriers (shown in red). Spatial overlap of increased amyloid-β burden was observed in frontal and temporal regions across two studies. Brain maps were created using Simple brain plot (Scholtens et al., 2021).

Four studies examined differences in cerebral amyloid-β (Aβ) deposition between *APOE ε4-*carriers and non-carriers (Fig. 4). Methodological differences were evident across studies, particularly in the handling of *APOE ε2-*carriers, who were either excluded, included within control groups, or not explicitly reported. Imaging resolutions also differed, with two studies reporting voxel sizes of ∼2 mm, while one reported a common spatial resolution of 8 mm.

Across most studies, greater cortical Aβ deposition was reported in *APOE ε4-*carriers compared with non-carriers, using Pittsburgh compound-B (PiB) (Mecca et al., 2018; Stonnington et al., 2020) or combined PiB and florbetapir imaging (Thirunavu et al., 2019). Increased Aβ burden was most consistently observed in frontal and association cortices, consistent with early cortical vulnerability. One study found increased Aβ deposition in *APOE ε4-*carriers across the averaged frontal, posterior cingulate, precuneus, lateral parietal, and lateral temporal cortices (*p =* 0.003) (Mecca et al., 2018). Another study combined the prefrontal cortex, precuneus, gyrus rectus, and lateral temporal regions, finding greater Aβ deposition in *APOE ε4-*carriers compared to non-carriers (β = 6.1, *p* = 0.0012) (Thirunavu et al., 2019). A separate study performed a voxel-wise analysis and found *APOE ε4-*carriers possessed greater Aβ deposition primarily in orbitofrontal, temporal, and temporal pole regions (*p* < 0.001) (Stonnington et al., 2020). Mean participant ages were similar across studies (range: 54–59 years) and all controlled for at least age and sex.

Only one study reported no group differences in Aβ deposition between *ε4-*carriers and non-carriers in either the precuneus or pooled cortical regions (Bussy et al., 2019). This null finding may be partially explained by the younger age of the sample (ε4 carriers: 37.2 years; non-carriers: 43.0 years). Supporting this interpretation, the same study reported a significant *APOE* **×** age interaction, with older but not younger individuals carrying the *APOE ε4* allele showing greater Aβ deposition. However, this interaction was no longer present when participants aged over 50 years were excluded, suggesting that age-related effects may have been driven primarily by older individuals within the sample.

Overall, *APOE ε4*-carriers show evidence of greater cortical Aβ deposition, in temporal and parietal cortices, compared to non-carriers, in healthy midlife cohorts, with all studies aptly controlling for key variables such as age and sex.

### 3.3 Magnetic resonance imaging (MRI)

Forty studies used MRI to examine brain structure in cognitively healthy mid-life *APOE ε4-*carriers and non-carriers (Table 2). Sample sizes varied considerably, ranging from single-site cohorts with as few as 24 participants, to large multi-site analyses including 1685 individuals. Structural alterations were most commonly assessed using region-of-interest (ROI) analyses or voxel-based morphometry, targeting key AD-relevant regions such as the hippocampus, as well as broader tissue segmentations including grey and white matter volumes. Evidence across studies was mixed, reflecting differences in sample size, analysis methods, and participant age.

**Table 2:** MRI study characteristics.

Author	Country	Sample Size	Age (±SD)	Genotype	Field Strength	Main Finding
Structural MRI						
(Kewei Chen et al., 2007)	USA	36	57.4 (3.6)	a,b	1.5 T	ε4 homozygotes had greater annualized decline in total brain volume compared to non-carriers (*p =* 0.03, *d =* 1.079).
(Mueller et al., 2008)	USA	28	48.1 (9.7)	a,b	4 T	Young ε4 carriers had no differences across any hippocampal subfield compared to non-carriers.
(Panizzon et al., 2010)	USA	375	55.9 (2.6)	a,b	1.5 T	ε4 carriers had decreased hippocampal volume compared to non-carriers (*p* < 0.05).
(Ready et al., 2011)	USA	23	55.8 (5.0)	2,b	1.5 T	ε4-carriers had lower WM volume compared to non-carriers (*p <* 0.05).
(Fennema-Notestine et al., 2011)	USA	382	55.5 (2.8)	1,2	1.5 T	ε3/ε4-group had a thinner bilateral superior frontal, right caudal midfrontal, and left rostral midfrontal regions (*p <* 0.05, *d =* −0.21 *to* − 0.3)*.* ε4-carriers associated with greater volumes in the fusiform gyrus (*p <* 0.05, *d =* 0.22).
(Chen et al., 2012)	USA	130	56.2 (4.7)	2,3,b	1.5 T	Lower grey matter in parietal, anterior cingulate, temporal, hippocampus, parahippocampal, and inferior temporal regions in a ε4-gene dose effect (*p <* 0.05).
(Alexander et al., 2012)	USA	24	38.1 (5.2)	1,a	1.5 T	ε4-carriers had lower grey matter network patterns in prefrontal, temporal, and parietal cortices (*F*_*(*2,21*)*_ = 8.11, *p* < 0.002).
(Donix et al., 2013)	Germany	26	55.3 (7.0)	a,b	3 T	ε4-carriers had significantly thinner left entorhinal cortex than right entorhinal cortex, compared to non-carriers (*p =* 0.002, *d =* 1.12).
(Protas et al., 2013)	USA	149	56.3 (4.6)	2,3,b	1.5 T	ε4-carriers show no differences in hippocampal volume compared to non-carriers.
(ten Kate et al., 2016)	Netherlands	290	57.7 (6.8)	a,b	3 T	ε4-carriers show no differences grey matter volume compared to non-carriers.
(Ritchie et al., 2017)	France	208	53.0 (6.5)	2,3,b	3 T	ε4-carriers show no differences in hippocampal volume compared to non-carriers.
(Stefaniak et al., 2018)	UK	160	51.6 (5.6)	2,3,b	3 T	ε4-carriers show no differences in white matter hyperintensity or cerebral microbleeds compared to non-carriers
(Cacciaglia et al., 2018)	Spain	553	57.9 (7.3)	2,3,b	3 T	ε4-carriers had lower grey matter volume in the right hippocampus, caudate, precentral gyrus, and cerebellar crus. APOE4 was also associated with greater grey matter volume in the right thalamus, left occipital gyrus, and right frontal cortex (*p <* 0.01).
(Bussy et al., 2019)	USA	162	38.8 (10.7)	2,b	3 T	ε4-carriers showed no volumetric differences compared to non-carriers.
(Salvadó et al., 2019)	Spain	575	58.0 (6.5)	2,3,b	3 T	ε4-carriers showed no associations with white matter hyperintensity burden compared to non-carriers.
(Stonnington et al., 2020)	USA	114	56.8 (4.4)	a,b	3 T	ε4-carriers show no differences in hippocampal volume compared to non-carriers.
(Vilor-Tejedor et al., 2020)	Spain	302	57.1 (5.7)	2,3,b	3 T	ε4-carriers show no differences in hippocampal subfield volume compared to non-carriers.
(Dounavi et al., 2020)	UK	180	51.8 (5.4)	a,b	3 T	ε4-carriers showed a lower molecular layer volume compared to non-carriers (*p* = 0.03, *d =* −0.44).
(Mole et al., 2020)	UK	165	55.7 (8.2)	2,3,b	3 T	ε4-carriers showed a reduced macromolecular proton fraction in the thalamus (*p* < 0.001, η_p_^2^ = 0.8).
(Sala-Vila et al., 2021)	Spain	340	57.6 (7.6)	a,b	3 T	ε4-carriers showed no differences in brain volume compared to non-carriers.
(Dounavi et al., 2022)	UK	600	51.2 (5.4)	2,3,b	3 T	ε4-carriers showed no differences in regional brain volume or hippocampal subfield volume, compared to non-carriers.
(Dounavi et al., 2024)	UK	1585	55.5 (6.8)	2,3,b	3 T	ε4-carriers showed no differences in brain volume or texture compared to non-carriers.
(Leclaire et al., 2024)	UK	128	55.5 (6.0)	2,3,b	3 T	ε4-carriers showed thinner left rostral middle frontal gyrus compared to non-carriers (*p <* 0.01).
(Laurell et al., 2025)	UK	1939	57.0 (7.3)	2,3,b	3 T	ε4-homozygotes had greater anterior-superior hypothalamic volume compared to ε4-heterozygotes (β = −2.23 *p* = 0.02) and non-carriers (β = −2.07, *p* = 0.02).

Diffusion MRI						
(Bendlin et al., 2010)	USA	135	57.8 (5.3)	2,3,b	3 T	ε4-carriers showed no differences in FA or MD compared to non-carriers.
(Westlye et al., 2012)	Norway	203	47.6 (14.9)	2,b	1.5 T	ε4-carriers showed greater RD and MD, in 12.5% and 18.5% of voxels respectively (*p* < 10^*−5*^, *d =* 0.79).
(Patel et al., 2013)	USA	203	46.7 (7.5)	a,b	3 T	ε4-carriers showed no differences in FA compared to non-carriers.
(Feis et al., 2019)	Netherlands	75	49.6 (10.4)	a,b	3 T	ε4-carriers had four clusters of reduced FA in *APOE ε4-*carriers - three in the right splenium of the corpus callosum and one in the right inferior fronto-occipital fasciculus (*t =* 3.19–4.19).
(Mole et al., 2020)	UK	165	55.7 (8.2)	2,3,b	3 T	ε4-carriers showed no differences in NODDI metrics compared to non-carriers.
(Mak et al., 2024)	UK	1954	57.0 (7.26)	a,b	3 T	ε4-carriers found the orientation dispersion index was higher in regions of older APOEε4-carriers in an APOE ε4 × age interaction (*p <* 0.05).

Perfusion MRI						
(McKiernan et al., 2020)	UK	162	51.9 (5.5)	a,b	3 T	ε4-carriers showed no differences in brain volume compared to non-carriers, but showed increased cerebral blood flow in left cingulate, left frontal, and parietal regions compared to non-carriers (*p <* 0.01).
(Dounavi et al., 2021)	UK	162	52.1 (5.3)	a,b	3 T	ε4-carriers showed regions of greater cerebral blood flow in the ACA and MCA compared to non-carriers (*p <* 0.02, β = 3.31–4.36).
(Alruwais et al., 2022)	Saudi Arabia	36	52 (4)	1,a	3 T	ε4-carriers showed no difference in blood brain barrier permeability, compared to non-carriers.
(Dounavi et al., 2023)	UK	275	51.3 (5.5)	2,3,b	3 T	ε4-carriers showed regions of greater cerebral blood flow compared to non-carriers (*p* < 0.01).
(Aamand et al., 2024)	Denmark	76	52.3 (12.0)	1,a	3 T	ε4-carriers showed regions of greater cerebral blood flow and lower capillary blood volume compared to non-carriers (*p* < 0.01).

Functional MRI						
(Trachtenberg et al., 2012)	UK	77	45.9 (4.3)	a,b	3 T	ε4-carriers show patterns of altered connectivity across four major networks: Anterior hippocampal network, posterior hippocampal network, auditory network (*p* < 0.05).
(Patel et al., 2013)	USA	203	46.7 (7.5)	a,b	3 T	ε4-carriers show patterns of decreased connectivity in the PCC and precuneus, and increased connectivity in select regions, however, results did not survive family wise error correction.
(Goveas et al., 2013)	USA	46	53.6 (5.7)	a,b	3 T	ε4 non-carriers had lower functional connectivity within the default mode network (DMN), lower anticorrelation and connectivity within the executive control network (ECN) and increased positive correlation in the salience networks (SN) in *APOE ε4-*carriers (*p <* 0.05*).*
(Li et al., 2014)	USA	46	53.6 (5.7)	a,b	3 T	ε4*-*carriers showed lower hippocampal functional connectivity within subcortical regions, frontal cortex areas, DMN, and the cerebellum (*p <* 0.05).
(Wink et al., 2018)	Netherlands	261	56.9 (6.7)	a,b	3 T	ε4-carriers show lower eigenvector centrality, a lower connectivity of key regions, in the visual cortex, compared to non-carriers (*p <* 0.05)
(Feis et al., 2019)	Netherlands	75	49.6 (10.4)	a,b	3 T	ε4-carriers showed no functional connectivity differences compared to non-carriers.

MR Spectroscopy						
(Oleson et al., 2020)	USA	122	49.2 (7.31)	a,b	3 T	ε4-carriers had greater glutamate concentrations in the cerebral cortex compared to non-carriers, in the presence of increased dietary polyunsaturated fat (*p* = 0.025).
(Jett et al., 2023)	USA	209	51 (6)	a,b	3 T	Lower ATP/PCr ratio in male *APOE ε4-*carriers compared with male non-carriers. Both female *APOE ε4-*carriers and female non-carriers compared with male non-carriers, within the frontal lobe (*p* = 0.046).

1 = ε3 homozygotes; 2 = ε4 heterozygotes; 3 = ε4 homozygotes; a = ε4 carriers; b = ε4 non-carriers.

#### 3.3.1 Volumetric analysis

##### 3.3.1.1 Global volumetric analysis

Thirteen studies (12 cross-sectional; 1 longitudinal) examined associations between *APOE ε4* genotype and global brain volumes using MRI, with sample sizes ranging from 38 to 1685 participants (Dounavi et al., 2024; Bussy et al., 2019; Dounavi et al., 2022; Feis et al., 2019; Goveas et al., 2013; Leclaire et al., 2024; Li et al., 2014; McKiernan et al., 2020; Ritchie et al., 2017; Sala-Vila et al., 2021; ten Kate et al., 2016; Kewei Chen et al., 2007; Ready et al., 2011).

Findings predominately reported no significant group differences between *APOE ε4-*carriers and non-carriers (Dounavi et al., 2024; Bussy et al., 2019; Dounavi et al., 2022; Feis et al., 2019; Goveas et al., 2013; Leclaire et al., 2024; Li et al., 2014; McKiernan et al., 2020; Ritchie et al., 2017; Sala-Vila et al., 2021; ten Kate et al., 2016), while one small cross-sectional study (*n* = 13) reported lower white matter volume in *APOE ε4-*carriers (*p <* 0.05) (Ready et al., 2011). A separate longitudinal study (*n* = 36) observed increased whole brain atrophy rates in *APOE ε4-*homozygotes compared to non-carriers (*p =* 0.03, *d =* 1.079) (Kewei Chen et al., 2007); however, the small sample size limits the reliability and generalisability of these findings. Overall, the current evidence does not support consistent *APOE ε4-*related differences in global brain volumes.

##### 3.3.1.2 Hippocampus and medial temporal lobe

The hippocampus has been a primary focus due to its early involvement in AD pathology and central role in memory. Thirteen studies assessed hippocampal volume in midlife cohorts with sample sizes ranging from 23 to 553 participants.

In studies assessing bilateral or unilateral hippocampal volume, most reported no significant main effect of *APOE ε4* genotype (sample size ranged from 23 to 302) (Protas et al., 2013; Bussy et al., 2019; Stonnington et al., 2020; Ritchie et al., 2017; Ready et al., 2011; Donix et al., 2013; Dounavi et al., 2020; Mueller et al., 2008; Vilor-Tejedor et al., 2020). Conversely, a small minority of studies identified lower hippocampal volumes in *APOE ε4-*carriers (Chen et al., 2012; Cacciaglia et al., 2018; Panizzon et al., 2010). In the first with a large cohort (*n* = 553), lower right hippocampal volumes were found in *APOE ε4-*homozygotes compared with all other *APOE ε-*allele combinations (*p <* 0.01) (ε2/ε2 and ε2/ε3, ε2/ε4, ε3/ε3, ε3/ε4) (Cacciaglia et al., 2018). Similarly, a study (*n* = 130) utilised a multi-modal partial least squares (MMPLS) approach to identify patterns across sets of images, reported lower bilateral hippocampal volumes among *APOE ε4-*carriers, alongside reductions in other cortical regions (Chen et al., 2012). The third study (*n* = 375) identified *APOE ε4-*carriers as having lower hippocampal volumes compared to non-carriers (*p <* 0.02). *APOE ε4-*carriers also displayed lower hippocampal volumes in interaction with lower testosterone levels (*p <* 0.05) (Panizzon et al., 2010). When considered alongside aging effects, the final study *(n* = 162) reported a significant *APOE* × age interaction, such that hippocampal volumes were only lower in older *APOE-*ε4 carriers (interaction *p =* 0.028). Overall, cross-sectional evidence of lower whole hippocampal volumes is inconsistent and requires further research with larger sample sizes.

Longitudinal evidence is limited. Two studies found no significant *APOE ε4-*related change in hippocampal volume over time. One 15-year follow-up (*n* = 114) observed no genotype specific longitudinal decline (Bussy et al., 2019), while the other study reported no differences at baseline or after two years (Stonnington et al., 2020).

High-resolution hippocampal subfield analyses provide more detailed insights on region-specific vulnerability. Five studies have employed hippocampal segmentation to better characterise early neurodegenerative changes: Two used an independently validated manual segmentation technique (Donix et al., 2013; Mueller et al., 2008), while three used FreeSurfer, an automated segmentation tool (Dounavi et al., 2022; Dounavi et al., 2020; Vilor-Tejedor et al., 2020).

Four studies; two employing manual segmentation (*n* = 26; *n* = 28) and two employing FreeSurfer segmentation (*n* = 430; *n* = 600), reported null findings across all hippocampal subfield volumes when comparing *APOE ε4-*carriers with non-carriers (Dounavi et al., 2022; Donix et al., 2013; Mueller et al., 2008; Vilor-Tejedor et al., 2020). One study (*n* = 178), combined T1-weighted MPRAGE with high-resolution coronal T2-weighted images and found *APOE ε4-*carriers had a lower molecular layer volume than non-carriers, which remained significant after adjusting for age (*p* = 0.03, Cohen's *d* = −0.44) (Dounavi et al., 2020). The molecular layer is the outermost layer of the hippocampus, above the granule cell layer in the dentate gyrus, and is a major input zone receiving perforant path projections from the entorhinal cortex, essential for learning, memory consolidation, and spatial navigation. A two-year follow-up analysis of the same cohort (*n* = 155) showed a reduction in molecular layer volume among *APOE ε4*-carriers; however, this effect did not survive multiple comparison correction (Dounavi et al., 2020).

Overall, total hippocampal volume appears largely preserved in cognitively healthy midlife *APOE ε4-*carriers. However, lower volumes in specific subfields, particularly the molecular layer, may represent early, subtle structural changes indicative of preclinical vulnerability, though these findings warrant replication in larger and more well-characterised cohorts.

##### 3.3.1.3 Cortical regions

In addition to the hippocampus, fifteen studies have examined early structural changes associated with *APOE ε4* across cortical grey matter regions*.* Ten studies (71%) found no significant volumetric differences in cortical grey matter volume across multiple regions of interest, with sample sizes ranging from 46 to 1585 participants (Dounavi et al., 2024; Bussy et al., 2019; Dounavi et al., 2022; Feis et al., 2019; Goveas et al., 2013; Li et al., 2014; McKiernan et al., 2020; Sala-Vila et al., 2021; Vilor-Tejedor et al., 2020; Mole et al., 2020). However, five studies reported that *APOE ε4-*carriers had lower volumes across multiple cortical regions.

The five studies that reported regional volumetric or thickness differences between *APOE ε4-*carriers differed across studies with varying sample sizes (*n* = 26–553). The largest study, conducted in Germany, identified reduced regional brain volumes in the right precentral gyrus, right caudate nucleus, and right cerebellar crus in *APOE ε4-*carriers compared to non-carriers (*p* < 0.05) (Cacciaglia et al., 2018). Similarly, a large cohort (*n* = 382) demonstrated thinner cortices in bilateral superior frontal, right caudal midfrontal, and left rostral midfrontal regions of *APOE ε4-*carriers, with a small effect size (*p* < 0.05, Cohen's *d* ranges from −0.21 to −0.30) (Fennema-Notestine et al., 2011). Using an MMPLS approach, a medium-sized study (*n* = 130) further revealed reduced grey matter volume across parietal, anterior cingulate, temporal hippocampal, parahippocampal, and inferior temporal regions, showing a gene-dose effect with increasing *APOE ε4*-allele number (*p* < 0.05) (Chen et al., 2012). In another moderately sized cohort (*n* = 128), lower volume in the left rostral middle frontal gyrus was observed in *APOE ε4-*carriers relative to non-carriers (*p* < 0.02) (Leclaire et al., 2024). The smallest study (*n* = 26), however, reported a markedly larger effect, with significantly thinner left entorhinal cortices compared to the right in *APOE ε4-*carriers compared to healthy non-carriers (11.3% volume difference, *p =* 0.002, *d =* 1.12) (Donix et al., 2013).

##### 3.3.1.4 Other subcortical regions

Three studies have also examined other subcortical regions outside of the hippocampus, with mixed findings. For example, one study (*n* = 553) reported lower caudate volumes in *APOE ε4-*carriers compared with non-carriers, despite examining multiple subcortical structures including the hippocampus, thalamus, caudate nucleus, hypothalamus, and cerebellar crus (*p* < 0.01) (ten Kate et al., 2016; Cacciaglia et al., 2018). Conversely, greater volumes have also been reported in the right medial thalamus (*p* < 0.01) (Cacciaglia et al., 2018), anterior–superior hypothalamus (β = −2.23, *p* = 0.02) (Laurell et al., 2025) and in the bilateral superior frontal cortices (*p* < 0.01) (Leclaire et al., 2024). While neurodegeneration is typically associated with widespread volume loss, these localised increases in brain volumes among *APOE ε4-*carriers may reflect specific responses to early AD pathology, including neuroinflammation, transient brain swelling in response to early amyloid deposition, or even developmental differences in brain structure. Overall, there is minimal evidence to suggest that other subcortical structures, outside of the hippocampus, show volumetric differences in healthy, midlife *APOE ε4-*carriers.

#### 3.3.2 White matter and cerebral microbleeds

Two European cohort studies, one from the PREVENT cohort (UK) and the other from ALFA (Spain), (*n* = 160 and *n* = 575) examined the effects of *APOE ε4* on white matter hyperintensities (WMHs) and cerebral microbleeds using MRI. No APOE effects were found in the number of white matter hyperintensities (Stefaniak et al., 2018; Salvadó et al., 2019), or cerebral microbleeds (Salvadó et al., 2019), measured with Fluid Attenuated Inversion Recovery (FLAIR) and Susceptibility-Weighted Imaging (SWI), respectively.

#### 3.3.3 Grey matter networks

One study applied Scaled Sub profile Modelling (SSM) to examine coordinated differences across grey matter volumes as opposed to looking at isolated regions (Alexander et al., 2012). Results indicated significantly lower grey matter network patterns in the prefrontal, temporal, and parietal cortices of *APOE ε4-*carriers compared to non-carriers, suggesting subtle, network-level atrophy or reorganisation (*F*_*(*2,21*)*_ = 8.11, *p* < 0.002). However, the relatively small sample size (*n* = 24) and the fact that SSM has not been widely applied in other studies, limit generalisability and highlights the need for replication.

#### 3.3.4 Texture-based morphometry

Texture-based morphometry evaluates voxelwise patterns of grey and white matter relative to neighbouring voxels, estimating features such as intensity randomness (entropy), differences between adjacent voxels (contrast), homogeneity, and voxel uniformity (energy). One study reported a small, nonsignificant trend toward lower homogeneity, higher contrast and entropy in *APOE ε4*-carriers (Dounavi et al., 2024). However, 553 of 1585 participants (35%) were excluded due to image artifacts or incidental findings, which may increase the risk of bias and lower validity of the evidence.

#### 3.3.5 Quantitative magnetization transfer

Quantitative magnetization transfer (QMT) measures the difference in magnetization between protons in free water and those bound to large macromolecules, allowing quantification of tissue macromolecular content. Unlike conventional MRI, which primarily measures signals from free water protons, QMT provides insights into the integrity of macromolecular structures such as myelin. A single UK study (*n* = 165) applied QMT to compare myelin content between A*POE ε4*-carriers and *APOE ε4* non-carriers, across 68 cortical and 14 subcortical regions (Mole et al., 2020). Using multivariate analyses of covariance, controlling for age, sex, and IQ, they found a lower macromolecular proton fraction in the left thalamus of *APOE ε4*-carriers compared with non-carriers *(p* < 0.001, η_p_^2^ = 0.8), suggesting lower myelin content and potential degeneration. Future work should aim to apply the robust statistical framework employed here to larger cohorts, enabling meaningful replication and validation of these findings. This would help clarify the broader utility of QMT, which remains difficult to establish from a single, moderately sized study alone.

#### 3.3.6 Diffusion MRI

Six studies have used diffusion MRI to investigate microstructural features in *APOE ε4*-carriers and non-carriers. Study characteristics varied considerably, including sample size (*n* = 75 to *n* = 1954 participants), diffusion encoding directions (12–90), b-values magnitude (0–6000), and number of b-values (1–6), contributing to study heterogeneity.

The findings from studies were inconsistent. Two studies reported no significant differences in fractional anisotropy (FA) across the whole brain between *ε4-*carriers and non-carriers (Westlye et al., 2012; Patel et al., 2013). In contrast, another study identified four clusters of reduced FA in *APOE ε4-*carriers - three in the right splenium of the corpus callosum and one in the right inferior fronto-occipital fasciculus (*t =* 3.19–4.19) (Feis et al., 2019), which may reflect demyelination or axon loss. Another study highlighted the importance of considering the combined effect of family history (FH) and *APOE ε4* on FA. Results revealed that higher combined genetic and familial risk was associated with lower FA, but how this effect was driven by FH, with no independent contribution from *APOE ε4* (Bendlin et al., 2010).

Other diffusion metrics showed partial differences in *APOE ε4-*carriers. Higher radial diffusivity (RD) and mean diffusivity (MD) were reported in *APOE ε4-*carriers compared to non-carriers, affecting 12.5% and 18% of voxels respectively, across multiple brain regions, potentially reflecting early neuroinflammatory processes (*p* < 10^*−*5^, *d =* 0.79) (Westlye et al., 2012).

Two studies applied Neurite Orientation Dispersion and Density imaging (NODDI) to examine tissue microstructure, including neurite density, degree of orientation (ODI), and free water fraction. In the largest study (*n =* 1954; mean age 57 years) NODDI metrics were extracted from occipital, parietal, temporal, frontal, cingulate, and insula regions. The study found no main effect of *APOE ε4* across any tested regions for any NODDI metrics. However, when including age in analysis, five regions (frontal, temporal, parietal, cingulate, insula) showed significant age **×** *APOE ε4-*interactions, with older *APOE ε4-*carriers displaying lower ODI values (*p <* 0.05) (Mak et al., 2024). This suggests that older *APOE ε4-*carriers exhibit lower cellular complexity as age increases, with the greatest effects in parietotemporal regions. However, this effect was not corroborated in a smaller study (*n* = 165, mean age = 55.7 years) with no significant *APOE ε4-*effect reported across any NODDI metric (Mole et al., 2020).

Overall, diffusion MRI evidence for *APOE ε4-*related microstructural changes in midlife is mixed, with some localised effects observed but with limited replication across cohorts.

#### 3.3.7 Perfusion MRI

Five studies (four cross-sectional; 1 longitudinal) investigated cerebrovascular alterations associated with *APOE ε4* using perfusion MRI, with sample sizes ranging from 36 to 375 participants with an average age of 52 years. Three studies applied arterial spin labelling (ASL) to assess cerebral blood flow (CBF) (McKiernan et al., 2020; Dounavi et al., 2021; Dounavi et al., 2023), one employed dynamic susceptibility contrast MRI to examine microvasculature CBF (Aamand et al., 2024), and one study employed dynamic contrast-enhanced MRI to evaluate blood-brain barrier (BBB) dysfunction and altered perfusion using a gadolinium-based contrast agent (Alruwais et al., 2022).

Several studies suggest greater CBF in *APOE ε4-*carriers compared with non-carriers. Increased perfusion was observed in territories supplied by the anterior cerebral artery (ACA) (*p* < 0.01) and middle cerebral artery (MCA) (*p* < 0.01) (Dounavi et al., 2021; Dounavi et al., 2023). Regionally, greater perfusion in *APOE ε4*-carriers was also reported bilaterally across cortical regions, with the strongest effects reported in the left cingulate and lateral frontal cortices (*p <* 0.01), although this study did not clearly report significance thresholds, specify all regions affected, or provide a table of results (McKiernan et al., 2020).

Converging with these findings, studies incorporating microvascular measures suggest that elevated CBF in *APOE ε4* carriers may coexist with altered capillary-level function. Using dynamic susceptibility contrast MRI, one study identified eleven regions of increased CBF and eleven regions of decreased capillary blood volume in *APOE ε4-*carriers across temporal, parietal, frontal, and occipital lobes. Six regions – including the cuneus, lingual gyrus, medial and middle gyri, middle temporal gyrus and cerebral white matter - showed the combination of increased CBF and reduced capillary blood volume (*p* < 0.01), suggesting impaired microvascular oxygen extraction rather than enhanced perfusion efficiency (Alruwais et al., 2022). Finally, one study assessed blood brain barrier permeability (*n =* 36), using a gadolinium-based contrast agent, however, no group differences were found across any brain regions.

The only longitudinal study, from the PREVENT-Dementia cohort, examined cognitively normal adults aged between 40 and 59 years (baseline *n* = 158, follow-up *n* = 125) (Dounavi et al., 2021). CBF and cerebrovascular resistance index (CVRi) were measured using ASL and corrected for brain atrophy and hematocrit. At baseline, *APOE ε4-*carriers showed higher CBF and lower CVRi in anterior and middle cerebral arteries compared to non-carriers, consistent with relative vasodilation. Over two years, hemodynamic changes did not differ between groups. However, genotype-specific associations emerged between longitudinal CBF changes and cognitive performance: increased CBF was linked to improved delayed recall in non-carriers and improved verbal fluency in ε4 carriers.

Overall, perfusion MRI findings suggest that *APOE ε4-*carrier status in midlife is associated with regionally increased CBF, possibly alongside alterations in microvascular structure and function. These findings are consistent with early cerebrovascular dysregulation and possible vascular inefficiency. Further longitudinal studies using both macro- and microvascular metrics are required to clarify the onset, trajectory, and functional significance of these differences.

#### 3.3.8 Functional MRI

Six studies utilised resting-state functional MRI (fMRI) to examine functional connectivity differences associated with *APOE ε4-*carrier status. Sample sizes ranged from 36 to 261 participants, aged between 44 and 56 years. Five studies used blood‑oxygen-level-dependent (BOLD) functional connectivity across resting state networks (Feis et al., 2019; Goveas et al., 2013; Li et al., 2014; Patel et al., 2013; Trachtenberg et al., 2012) while one study applied eigenvector centrality (EC) analysis to characterise network-level connectivity (Wink et al., 2018). Methodological approaches and reported outcomes varied substantially across studies.

Evidence from one study suggests both reduced and increased functional connectivity in *APOE ε4-*carriers within the auditory, anterior, and posterior hippocampi, left frontoparietal, and lateral visual networks, compared with non-carriers (*p* < 0.05) (Trachtenberg et al., 2012). Unexpectedly similar effects were also observed in *APOE ε2-*carriers, suggesting that *APOE-*related connectivity differences may not be specific to AD risk (Trachtenberg et al., 2012).

Another study identified a trend toward reduced connectivity in the posterior cingulate and precuneus in *APOE ε4-*carriers, alongside increased connectivity in the superior temporal gyrus, supramarginal gyrus, insula, thalamus and caudate compared with *APOE ε3* heterozygotes (Patel et al., 2013). Participants in this study were younger than those in other cohorts (mean age: 42 years). In contrast, region-to-network analyses found no significant connectivity differences between *APOE ε3* homozygotes and *APOE ε4-*carriers across the anterior, posterior and inferior default mode networks or the anterior and posterior salience networks (Feis et al., 2019).

Studies employing alternative voxel-based morphometry (VBM) analysis reported additional differences. One study found lower functional connectivity within the default mode network (DMN), lower anticorrelation and connectivity within the executive control network (ECN) and increased positive correlation in the salience networks (SN) in *APOE ε4-*carriers, suggesting altered network coordination and decoupling between the DMN and ECN (*p <* 0.05) (Goveas et al., 2013). When analysing the hippocampal network, *APOE ε4-*carriers showed lower hippocampal functional connectivity within subcortical regions, frontal cortex areas, DMN, and the cerebellum (*p <* 0.05) (Li et al., 2014).

Finally, one study employing EC (*n* = 261) reported lower centrality in the bilateral superior lateral occipital lobes and the dorsal PCC in *APOE ε4-*carriers compared to non-carriers, indicating reduced hubness and network efficiency (Wink et al., 2018). However, interpretation of these findings is limited by restricted slice coverage and the confinement of the analysis to the DMN because of scanning time constraints.

Overall, evidence from resting-state fMRI remains mixed. While some studies report *APOE ε4*-dependent alterations in functional connectivity, others describe comparable connectivity patterns in *APOE ε2-*carriers or fail to observe consistent *APOE ε4*-specific effects.

#### 3.3.9 Magnetic resonance spectroscopy (MRS)

Two studies from the USA used MRS to examine cerebral metabolic differences associated with *APOE ε4-*status (Oleson et al., 2020; Jett et al., 2023). Using a 6 cm isotropic voxel in the occipitoparietal grey matter, one study (*n* = 122) reported significantly higher cerebral glutamate levels in *APOE ε4-*carriers, compared to non-carriers (*p* = 0.025); however, the primary aim of this study was to investigate the effects of dietary polyunsaturated fatty acids on memory performance rather than *APOE* genotype (Oleson et al., 2020). An independent study using a 3 cm isotropic voxel across the whole brain (*n* = 209) assessed high-energy phosphate metabolites, including adenosine triphosphate (ATP), phosphocreatine (PCr), inorganic phosphate (Pi), and membrane phospholipids, stratified by sex and *APOE* status. Results identified significantly lower ATP/PCr ratio in male *APOE ε4-*carriers compared with male non-carriers, as well as in both female *APOE ε4-*carriers and female non-carriers compared with male non-carriers, within the frontal lobe (multi-variable adjusted *p* = 0.046) (Jett et al., 2023). Additionally, female ε4 carriers showed a lower PCr/ATP ratio in the temporal lobe relative to male non-carriers (*p* < 0.010).

Overall, these findings suggest that *APOE ε4-*carrier status is associated with regional and sex-specific alterations in cerebral metabolic profiles; however, the limited number of studies restrict the ability to draw firm conclusions.

## 4 Discussion

This systematic review synthesised evidence from 46 neuroimaging studies examining associations between *APOE ε4-*allele carrier status and brain outcomes in cognitively healthy midlife adults. Most studies compared *APOE ε4-*carriers to *ε4* non-carriers, with a small subset incorporating gene-dose analyses. Overall, reporting quality was variable: effect sizes and power calculations were frequently unreported, *APOE* ε2-carriers were often included within control groups*,* few studies employed longitudinal analyses, and none were conducted in low- or middle-income countries. Together, these limitations highlight a critical gap between the breadth of neuroimaging research and the limited availability of high-quality, methodologically rigorous evidence characterising *APOE ε4-*effects in healthy midlife populations. These findings underscore the need for harmonised methodologies, standardised reporting, and larger, more diverse cohorts to improve reproducibility and validity of the current evidence base.

Across imaging modalities, the most consistent associations with *APOE ε4-*carrier status were observed in PET imaging of Alzheimer's disease pathology. Three of the four amyloid PET studies reported greater Aβ deposition in *APOE ε4-*carriers compared with non-carriers, particularly across frontal and parietal regions. The single null finding may reflect the younger age of the cohort (late 30's/early 40s), suggesting that amyloid deposition in *APOE ε4*-carriers may begin to emerge by the early 50s. FDG-PET studies similarly identified lower glucose metabolism within the posterior cingulate (identified in six of eight studies) and parietal cortex (identified in five of eight studies), mirroring metabolic alterations commonly seen in clinical AD (Salmon et al., 2024). These convergent findings suggest that altered amyloid burden and glucose metabolism may be detectable long before clinical symptoms emerge, positioning PET markers as some of the earliest *in vivo* indicators of AD risk. Although PET imaging is constrained by cost, scanner availability, and the ethical considerations associated with the use of radioactive tracers in healthy people, the robustness of these findings across heterogeneous methods supports its utility in identifying preclinical pathology.

An important gap identified by this review is the absence of tau-PET studies in cognitively normal midlife populations. Although plasma p-tau217 and tau-PET have been associated with subsequent cognitive decline in cognitively unimpaired adults (Ossenkoppele et al., 2025), and the relationship between *APOE* ε4 and tau pathology appears to be largely mediated by amyloid-β (Cicognola et al., 2025), these studies did not specifically examine tau burden in cognitively normal midlife cohorts aged ≤60 years who were at genetic risk of AD. Given the association between tau accumulation and cognitive decline (Bejanin et al., 2017), investigating tau pathology during midlife may help clarify when *APOE* ε4-associated neurobiological changes first emerge and how they interact with amyloid positivity over time. By identifying the lack of studies examining *APOE* ε4-associated tau burden in midlife, this review highlights a distinct gap in the literature and an important priority for future research.

MRI findings were less consistent, with studies often underpowered or using novel methods which have not been replicated in other cohorts. Structural MRI generally revealed no large-scale differences in whole brain, grey matter, or white matter volumes in *APOE ε4-*carriers, consistent with the expectation that macrostructural degeneration is primarily a feature of later aging and AD (Bottino et al., 2002; Du et al., 2001; Elshafey et al., 2014; Fujita et al., 2023; Mueller et al., 2010; Pennanen et al., 2004; Xia et al., 2025). More specific analyses of the hippocampal subfields suggest that subtle volumetric reductions may occur in regions such as the molecular layer (Dounavi et al., 2020), although this finding was not replicated in other studies, and requires further validation in larger, well-characterised cohorts. Some studies reported higher volumes in other subcortical regions in *APOE ε4-*carriers, which may reflect neuroinflammatory responses, compensatory mechanisms, or developmental differences causing increased volume in specific brain regions rather than degeneration (Leclaire et al., 2024; Cacciaglia et al., 2018; Laurell et al., 2025). However, this claim is hypothetical requiring longitudinal evidence, paired with inflammatory markers, to elucidate the mechanisms that cause greater brain volumes. There was no evidence to suggest an *APOE* effect on the burden of white matter hyperintensities or the presence of cerebral microbleeds (Stefaniak et al., 2018; Salvadó et al., 2019), indicating that overt small-vessel cerebrovascular pathology may not yet be evident by midlife, and suggests that this pathology might represent downstream pathological damage as opposed to an early marker of preclinical disease.

Diffusion MRI studies were highly variable in methodology, with variations in gradient strength, number of diffusion-weighted directions, and post-processing models. We found no robust differences in fractional anisotropy (FA) and only isolated reports of altered mean diffusivity (MD) and radial diffusivity (RD). In contrast, diffusivity measures are consistently reported to be higher in AD and MCI populations across white matter (Lv et al., 2025) and grey matter regions, including the hippocampus (Müller et al., 2005), amygdala and posterior cingulate cortex (Rose et al., 2008). Similarly, FA is reliably lower in AD and MCI across multiple brain regions, including the thalamus and parietal white matter (Rose et al., 2008), as well as regions of the corpus callosum (Lv et al., 2025). In this review, we highlight one large NODDI study offered some evidence that older *APOE ε4-*carriers show reduced neurite orientation dispersion index across frontal, temporal, parietal, cingulate and insula brain regions, indicating altered microstructural complexity, although this finding was not replicated in a smaller study. Future research should clarify whether microstructural degeneration occurs in midlife populations, and whether any such changes are driven primarily by age-related processes, genetic risk, or some other combination of factors.

Perfusion MRI studies consistently reported higher regional cerebral blood flow in *APOE ε4*-carriers, in contrast to the lower perfusion typically seen in AD patients (Yu et al., 2024; Zuin et al., 2024). One possible explanation is that compensatory mechanisms elevate CBF in younger *APOE ε4*-carriers, before increasing arterial stiffness and vascular dysfunction contribute to a subsequent decline in CBF in AD patients (Rivera-Rivera et al., 2021). Future research should aim to identify when CBF begins to increase in *APOE ε4-*carriers and the factors driving the transition from elevated to reduced CBF across the disease trajectory. Functional MRI studies of resting-state connectivity report complex and heterogeneous patterns, particularly within the default mode network, with no consistent signature in midlife carriers, whereas reductions in connectivity are consistently observed in MCI and AD (Sperling, 2011). MR spectroscopy data are limited, with isolated reports of altered metabolite ratios suggesting possible sex-specific differences (Sheikh-Bahaei et al., 2024), but small study numbers and methodological heterogeneity preclude definitive conclusions.

The risk-of-bias assessment demonstrated that the handling of *APOE* ε2 carriers was frequently unclear or inconsistent. Specifically, 35 of the 46 studies (76%) included participants carrying an *APOE* ε2 allele, excluded *APOE* ε2 carriers only from the *APOE* ε4-carrier group, or identified *APOE* ε2 carriers without clearly reporting whether they were included in the analyses. Given evidence that *APOE* ε2 is associated with lower AD risk relative to *APOE* ε3 and *APOE* ε4, combining ε2 and ε3 carriers within a single comparison group may introduce heterogeneity and influence estimates of *APOE* ε4-associated effects. Importantly, the interpretation of risk associated with each *APOE* allele depends on the reference genotype selected; *APOE* ε3 may appear to confer increased risk when compared with *APOE* ε2, whereas *APOE* ε2 appears protective when *APOE* ε3 is used as the reference (Williams et al., 2026). This does not establish *APOE* ε3 as an independently pathogenic allele but highlights the importance of clearly defining genotype groups. Future neuroimaging studies should report the inclusion and distribution of *APOE* ε2 carriers and, where sample size permits, analyse ε2, ε3, and ε4 genotypes separately. Greater attention to *APOE* ε2 may also provide insight into mechanisms of neurobiological resilience during midlife.

The assessment of primary health-related confounders, particularly cardiovascular risk factors and other medical comorbidities, was inconsistent across studies in both reporting and the analyses conducted. Across all modalities, only 21/46 studies described vascular risk factors within their population. For example, among the two studies examining white matter hyperintensity burden, one reported detailed vascular risk measures including CAIDE score, BMI, hypertension, hypercholesterolaemia, and physical activity, whereas the other provided little information regarding vascular risk factors. Surprisingly, neither study adjusted for these vascular risk factors or considered how they may contribute to imaging outcomes. However, we also note that the interpretation of vascular risk factor adjustment in this context is complex; these variables may not represent traditional confounders of the relationship between APOE ε4 carriership and neuroimaging outcomes. Instead, factors such as hypertension, hypercholesterolaemia, and broader cardiovascular risk may lie on the causal pathway between *APOE* genotype and brain structure or function. Consequently, adjustment for these variables may attenuate genuine *APOE* ε4-associated effects and could contribute to variability in findings across studies. This methodological issue should be considered when interpreting the impact of vascular risk factor adjustment within the reviewed literature.

The quantity of hypothesis-driven research was low across multiple modalities. Fluorodeoxyglucose PET (2/7 studies) and structural MRI (11/28 studies) contained the lowest proportion of hypothesis-driven research. Where hypotheses were specified, fluorodeoxyglucose PET studies typically predicted regionally reduced glucose metabolism, while structural MRI studies commonly hypothesised smaller volumes in brain regions considered vulnerable to AD pathology. Conversely, amyloid-β PET (3/4 studies) and functional MRI (4/6 studies) contained the highest proportions of hypothesis-driven research. Amyloid-β PET studies generally hypothesised that *APOE ε4*-carriers would exhibit greater amyloid deposition, particularly within regions vulnerable to later AD pathology, whereas functional MRI studies commonly predicted hyperexcitability during resting-state activity or altered connectivity within the default mode network and other relevant brain networks, with changes hypothesised to reflect either early network dysfunction or compensatory processes. Taken together, the limited use of *a priori* hypotheses complicates interpretation of the *APOE* ε4 neuroimaging literature. Reliance on exploratory analyses may increase heterogeneity, as studies vary in their selected outcomes, analytical methods, and statistical contrasts. Inconsistencies across studies may therefore reflect methodological differences as well as genuine biological variation associated with *APOE* ε4 status.

Many studies also lacked rigorous power analyses, with only three reporting an *a priori* power calculation, and effect size reporting was often absent, limiting the feasibility of meta-analyses. While the majority of studies (24/46) controlled for sex, relatively few examined sex-specific effects. Investigating sex differences is critical in the context of AD, as women have a higher lifetime risk of AD (Riedel et al., 2016) and may exhibit distinct patterns of pathological progression (Smith et al., 2020). Finally, all identified studies were conducted exclusively in high-income countries, primarily in the United States and Europe. As a result, findings may not be generalisable to populations in low-and-middle-income countries (LMICs), with geographical differences in *APOE ε4* allele frequency in healthy individuals (Wang et al., 2021) and AD patients (Osuntokun et al., 1995). This is a notable limitation, as environmental and sociodemographic factors such as education, early-life adversity, and infectious exposures may interact with genetic risk in ways that remain underexplored.

## 5 Conclusion

This systematic review has comprehensively evaluated the impact of the *APOE e4* allele on neuroimaging outcomes in healthy midlife individuals. PET evidence consistently identifies greater amyloid deposition and lower glucose metabolism among midlife *APOE ε4*-carriers, while MRI findings most reliably indicate higher cerebral blood flow. In contrast, other MRI-derived structural, microstructural, functional, and metabolic findings remain limited and largely inconclusive. This review also highlights how the majority of studies do not adequately account for *APOE ε2* status, which may bias estimates of *APOE ε4-*related associations with neuroimaging outcomes. Future research should prioritise adequately powered longitudinal studies in diverse populations, use harmonised imaging protocols with detailed and transparent reporting, and incorporate sex-stratified analyses to clarify how APOE genotype influences early brain changes and, ultimately, Alzheimer’s disease risk.

## CRediT authorship contribution statement

**Robert C. Davis:** Writing – review & editing, Writing – original draft, Visualization, Validation, Software, Resources, Methodology, Investigation, Formal analysis, Data curation, Conceptualization. **Louisa E. Wood:** Data curation. **Lily Wilkes:** Data curation. **Hannah L. Chandler:** Writing – review & editing, Methodology, Formal analysis. **Emma L. Anderson:** Writing – review & editing, Validation, Supervision, Methodology, Investigation, Conceptualization. **Lucy V. Hiscox:** Writing – review & editing, Visualization, Validation, Supervision, Software, Resources, Project administration, Methodology, Investigation, Funding acquisition, Formal analysis, Data curation, Conceptualization.

## Funding

This work was supported by the 10.13039/100010269Wellcome Trust [grant number: 226420/Z/22/Z]. The funders had no role in study design, decision to publish, or preparation of the manuscript.

## Declaration of competing interest

The authors declare that they have no known competing financial interests or personal relationships that could have appeared to influence the work reported in this paper.

## Data Availability

Data will be made available on request.
