# Supplementary material for: Impact of apolipoprotein Ε4 (APOE ε4) on neuroimaging outcomes in cognitively healthy midlife adults: A systematic review
Source: Neuroimage Clin. 2026 Jul 15;51:104035. doi: 10.1016/j.nicl.2026.104035 (PMC13445528; doi:10.1016/j.nicl.2026.104035)
Supplement: Supplementary file 1 [file mmc1.docx]

**Impact of Apolipoprotein Ε4 (*APOE ε4*) on Neuroimaging Outcomes in Cognitively Healthy Midlife Adults: A Systematic Review**

Robert C Davis^1*^, Louisa E Wood^1^, Lily Wilkes^1^, Hannah L Chandler^2^, Emma L Anderson^3^, Lucy V Hiscox^1^

^1^Cardiff University Brain Research Imaging Centre (CUBRIC), School of Psychology, Cardiff University, Maindy Road, Cathays, Cardiff, CF24 4HQ, UK.

^2^Cardiff University Brain Research Imaging Centre (CUBRIC), School of Physics and Astronomy, Cardiff University, Maindy Road, Cathays, Cardiff, CF24 4HQ, UK.

^3^Division of Psychiatry, University College London, Tottenham Court Road, London W1T 7NF, UK.

*****Corresponding Author

**Appendix S1**

**Supplementary Table S1**

Search strategy as used in MEDLINE. The search strategy is structured to identify papers explicitly containing variations of *APOE* (search lines 1-4), disease status and biomarkers (search lines 5-13), population health details (search lines 14-21), and neuroimaging modalities (search lines 22-35).

| 1. | ApoE*.tw. |
| --- | --- |
| 2. | apolipoprotein*.tw. |
| 3. | Apolipoprotein E4/ |
| 4. | 1 or 2 or 3 |
| 5. | dementia biomarker*.tw. |
| 6. | AD.tw. |
| 7. | Alzheimer's disease.tw. |
| 8. | Amyloid.tw. |
| 9. | Alzheimer's disease biomarker*.tw. |
| 10. | ad biomarker*.tw. |
| 11. | Amyloid beta-Peptides/ |
| 12. | dementia.tw. |
| 13. | 5 or 6 or 7 or 8 or 9 or 10 or 11 or 12 |
| 14. | prodromal.tw. |
| 15. | preclinical.tw. |
| 16. | asymptomatic.tw. |
| 17. | healthy.tw. |
| 18. | Midlife.tw. |
| 19. | middle-age*.tw. |
| 20. | prodromal symptoms/ |
| 21. | 14 or 15 or 16 or 17 or 18 or 19 or 20 |
| 22. | Magnetic Resonance Imaging/ |
| 23. | magnetic resonance imaging.tw. |
| 24. | MRI.tw. |
| 25. | positron emission tomography.tw. |
| 26. | PET.tw. |
| 27. | neuroimaging.tw. |
| 28. | brain imaging.tw. |
| 29. | computed tomography.tw. |
| 30. | CT.tw. |
| 31. | Positron-Emission Tomography/ |
| 32. | exp Neuroimaging/ |
| 33. | Tomography, X-Ray Computed/ |
| 34. | Magnetoencephalography/ |
| 35. | 22 or 23 or 24 or 25 or 26 or 27 or 28 or 29 or 30 or 31 or 32 or 33 or 34 |
| 36. | 4 and 13 and 21 and 35 |
